# Thematic Analysis of Smoking Cessation and Future Cessation Interventions for Cancer Survivors: Convenience Sampling Study

**DOI:** 10.2196/76792

**Published:** 2025-10-23

**Authors:** Kinsey Pebley, Tianna Williams, Kathryn Moody, Alana M Rojewski

**Affiliations:** 1 Department of Public Health Sciences, Medical University of South Carolina, Charleston, SC, United States; 2 Hollings Cancer Center, Medical University of South Carolina, 86 Jonathan Lucas St, Charleston, SC, 29425, United States, 1 843-779-5794

**Keywords:** smoking, smoking cessation, cancer, video, video intervention

## Abstract

**Background:**

Smoking after a cancer diagnosis has significant health consequences, and there are substantial benefits if cancer survivors cease smoking. However, there are few smoking cessation interventions for cancer survivors that have been effective, and they are highly resource intensive. Thus, novel low resource cessation interventions are needed. One such intervention modality that has not been tested among cancer survivors is using pre-recorded videos to deliver information about skills and resources to achieve smoking cessation.

**Objective:**

This study aimed to assess barriers to smoking cessation, interest in a video-based smoking cessation intervention, and video content preferences (eg, topic, video length, age and gender of video presenter, presenter type [peer, medical professional]) among individuals with cancer who smoke cigarettes.

**Methods:**

Participants were recruited from a cancer center in the southeast United States that houses an opt-out tobacco treatment program in which tobacco treatment specialists proactively call all patients who have a current smoking status in their medical record. Patients were informed about the study, and their contact information was sent to the study team. Verbal consent was obtained from individuals who were cancer survivors, who were currently smoking, and who agreed to participate; semi-structured qualitative interviews were conducted (n=10). Participants were asked questions about smoking history, previous quit attempts, barriers to quitting smoking, previous experience with and openness to watching videos about quitting smoking, and participant preferences related to video content and presentation. Interviews were transcribed and coded by two reviewers, and a codebook was developed. A thematic analysis was then conducted.

**Results:**

Results indicated that all participants had previously tried to quit smoking, but other substance use (5/10; 50%), negative affect (eg, depression [4/10; 40%], anxiety [1/10; 10%], stress [2/10; 20%], and loneliness [1/10; 10%]), the social environment (eg, being around others who smoke [2/10; 20%] and risky social situations [2/10; 20%]), and habits surrounding smoking were significant barriers for cessation. All participants were open to watching smoking cessation videos and expressed a desire to see a peer with lived experience in the videos. Most (8/10; 80%) participants did not have preferences related to the age or gender of video presenters. Many participants had difficulty articulating content preferences for the videos.

**Conclusions:**

Cancer survivors who smoke may not be aware of their knowledge gaps related to smoking cessation, and videos may be an acceptable way to provide evidence-based information to fill knowledge gaps.

## Introduction

Smoking remains the leading cause of preventable death in the United States, and smoking cessation is critical to improving health [1]. The health consequences of smoking are exacerbated after a cancer diagnosis, as cancer survivors (defined as any living person who has received a cancer diagnosis) who smoke are at increased risk of morbidity and mortality, while those who quit have improved treatment outcomes and survival [2-8]. However, an estimated 11.4% of survivors continue to smoke after diagnosis despite the health risks and impact to their cancer treatment outcomes [9]. Thus, there is significant room for improvement related to smoking cessation among cancer survivors.

Numerous smoking cessation interventions for cancer patients have been developed and tested, yet the majority have not resulted in increased smoking cessation rates compared to control interventions. One review found that 36 smoking cessation interventions for cancer survivors were tested by year 2020, and only 4 of these interventions resulted in statistically significant abstinence rates compared to control conditions [10]. Intervention strategies included methods such as contingency management and intensive counseling [10], meaning these interventions were also resource-intensive and required significant monetary and staff resources to deliver. Such interventions may not be feasible for under-resourced clinics, individuals living in rural areas without access to dedicated tobacco treatment services, or individuals who are interested in quitting on their own. Thus, there is a need for novel, effective, and less resource-intensive smoking cessation interventions for cancer survivors.

One intervention modality that has not been explored in the context of smoking cessation among cancer survivors, to our knowledge, is the delivery of smoking cessation content via video. Online informational videos are easily accessible via websites such as YouTube and thousands of online videos on these websites address smoking cessation. However, many of these videos do not depict evidence-based smoking cessation strategies [11]. Video-based interventions hold promise for a variety of other health considerations (eg, diabetes, chronic obstructive pulmonary disease, HIV, etc) by improving patient knowledge, facilitating health behavior changes, and increasing self-efficacy. However, many video intervention studies have been found to be at moderate to high risk of bias [12], and video interventions in the context of smoking cessation have not been as widely tested. One study conducted in the Netherlands found that a video intervention produced significantly higher long-term abstinence rates than a text-based intervention or generic advice [13]. Another study among individuals experiencing mental illness also found that a smoking cessation video intervention was associated with an increase in patient knowledge about quitting, and that this intervention was acceptable to patients [14]. Thus, video interventions for smoking cessation are promising, but there is a need for more work in this area across different populations.

The current study used semi-structured interviews with cancer survivors who smoke cigarettes to assess previous smoking cessation experiences, barriers to smoking cessation, interest in a novel video-based smoking cessation intervention, and preferences for video content and video presenter characteristics. The study was exploratory in nature and aimed to learn more about how videos should look (eg, who is presenting the information) and what survivors would like to learn more about. This qualitative study was planned to act as a guide to begin the creation of a video-based smoking cessation intervention tailored to the unique needs of cancer survivors.

## Methods

### Participants and Recruitment

Participants were recruited from the National Cancer Institute-designated Hollings Cancer Center at the Medical University of South Carolina, which is an academic medical center located in the southern United States. The cancer center houses an opt-out tobacco treatment program where all individuals receiving care at the cancer center who are designated as smoking in their medical chart receive a phone call to offer tobacco treatment services by a tobacco treatment specialist (TTS). The TTSs were asked to inform all of their patients that there was a study that they may be eligible for, reflecting a convenience sampling approach. The TTSs provided the patient’s contact information to the study team if they were interested in receiving more information. Inclusion criteria for participation included being 18 years or older, diagnosed with cancer (self-reported), and currently smoking cigarettes (self-reported). Exclusion criteria included not being able to speak English and no access to a telephone.

### Procedures

Results are reported following the Standards for Reporting Qualitative Research (SRQR) guidelines [15]. Semi-structured qualitative interviews were conducted by the primary investigator (PI, author KP) via telephone with participants using an interview guide created to lead the conversation related to patient experiences, barriers, and preferences (see Multimedia Appendix 1). Semi-structured interview methods were selected to allow for flexibility in the discussion, while also allowing for consistent gathering of data related to points of interest (eg, patient experiences and preferences). Sample questions from the interview guide include “What information would you like to learn more about if you were to watch videos about quitting smoking?” and “Do you think being able to watch videos on your personal device or computer about how to quit smoking could be helpful?” All data were collected via participant interview.

Interviews were conducted until thematic saturation was achieved (ie, no new ideas were discussed, and ideas participants brought up were repetitive), and the average duration was 20 minutes and 30 seconds.

Participants were asked questions about smoking history, previous quit attempts, barriers to quitting smoking, previous experience with and openness to watching videos about quitting smoking, and participant preferences related to video content and presentation. This included asking preferences about the perceived characteristics of people they would like to see present the content (eg, doctor versus peer, man or woman, older or younger person, etc). Interviews were recorded. They were then transcribed by two individuals (a trained medical student and a trained Master of Public Health [MPH] student[TW]) and coded by two coders (the PI [KP] and MPH student [TW]) based on an iteratively developed codebook. Specifically, recurring themes and ideas were identified by the PI and trained MPH student and listed as codes. These codes were refined and new codes were added as the PI and MPH student continued to review interview transcriptions until new themes and ideas were no longer identified. Coders had 86.5% agreement for the original codings, and a trained third coder (research assistant [KM]) was brought in to resolve discrepancies by coding the portion of the interview in which there was disagreement. An inductive thematic analysis was then conducted by the PI. Software was not used to facilitate the analysis.

### Ethical Considerations

Study procedures were approved by the Medical University of South Carolina Institutional Review Board (Pro00131304). Patients were called by the PI and given information about the study and provided verbal consent to participate prior to the interview. Given the low risk of the study, a waiver of written consent was granted by the Medical University of South Carolina Institutional Review Board. Participants received a US $30 Amazon gift card via email for their time. Data were deidentified, as identifying information was redacted in transcriptions.

## Results

### Participant Characteristics and Smoking History

** **The study team received information for 17 patients who were then contacted. One patient was ineligible (not actually diagnosed with cancer) and 6 could not be reached. Ten participants were interviewed, and 50% (5/10) reported White racial identity, 30% (3/10) reported Black racial identity, and 10% (1/10) reported biracial identity; 1 participant did not report their race. Sixty percent were women and 40% were men. The mean (SD) age was 55.9 (10.6) years (range 35‐72 years). Demographics of the current sample were similar to those of individuals who participate in the cancer center’s Tobacco Treatment Program (40% Black, 60% women, mean age of 58.8 y) [16]. Cancer diagnoses varied considerably and included breast, cervical, lung, bone, liver, prostate, thyroid, and brain cancers, with half of the participants reporting multiple cancers. Most (7/10) did not know their disease stage. All participants started smoking before age 18 years. The modal number of cigarettes smoked was 20 cigarettes per day (range: 2‐30).

### Methods for Quitting

** **All participants had made attempts to quit smoking previously. Five out of 10 participants had previously used nicotine patches, 2/10 had used nicotine gum, 2/10 had tried varenicline, and 2/10 tried to quit with no aid (ie, cold turkey). Only 1 participant had tried counseling before, and 1 had called a Quit Line.

### Barriers to Quitting

#### Substance Use

Half (5/10) of the participants endorsed other substance use as a barrier to quitting. Four participants reported that alcohol was a barrier, and 1 participant reported that the previous use of another undisclosed drug had previously been a barrier.  

There are two things that trigger it: the coffee and the drinking.

[Participant 10]

The first time I tried to quit, I had quit for three weeks and then we went out drinking for my 21^st^ birthday, and I started back.

[Participant 8]

#### Negative Affect

Another commonly reported barrier to quitting included negative affect, such as depression (4/10), anxiety (1/10), stress (2/10), or loneliness (1/10).

It’s just that today was my last day of work. I had a hard day and this [is] just depression not being able to go back to work [due to the cancer diagnosis].

[Participant 4]

…In between me and the treatments and being extra depressed and lonely and all of this other stuff going on with me, it’s been kinda hard to wanna focus on something different.

[Participant 3]

#### Social Environment

Four of the 10 participants also reported that being around others who smoked (2/4) or being in risky social situations (2/4) was a barrier to quitting and prompted relapses in the past.

*I was living with my brother and he was a smoker.* [Participant 5]

*And what happened when you went back to smoking? Is there something in particular that made you go back?* [Interviewer]

*Being around people that smoked*. [Participant 4]

#### Habits

Participants also reported different specific habits or daily routines related to smoking that acted as a barrier to quitting, such as coffee consumption.

*Make the coffee, let the dog out, light a cigarette. The hardest part is like, um, old behavior and old, uh, routines. Uh, like after I ate or getting up with a coffee in the morning. Um, when I drank alcohol, all that. It pushed me more to want a cigarette.* [Participant 1]

### Past Video Experience and Openness to Watching Videos

 Participants were asked if they had ever previously watched videos about quitting smoking, and if they would be open to watching videos in the future. Only 3/10 participants had seen videos about quitting smoking previously. Two participants reported that these videos were commercials; one of these participants stated she had seen commercials for nicotine replacement therapy such as nicotine gum, while the other did not identify the types of commercials they had seen. The third participant who reported seeing videos about quitting smoking stated these videos provided information about how to quit, such as strategies to distract. All participants said they were open to watching videos.

### Video Presentation and Content

Most participants also reported not having a preference related to gender (8/10; 2 did not respond to related to gender preferences) or age (8/10; 2 did not respond related to age) of the video presenter. Two participants reported that they would not want to see a younger adult delivering information in the videos. Some participants endorsed several different people they would be open to see presenting information (eg, peer with lived experience or doctor), but the majority endorsed wanting to see a peer with lived experience (8/10). 

When asked what information they would like to receive in the videos, many participants had a difficult time generating ideas and there was a wide variety of responses. However, several participants suggested behavioral strategies to avoid smoking, including finding alternative activities (3/10), getting rid of cigarettes (2/10), and replacing the habit of smoking with another activity (2/10).

*Techniques to replace a habit I guess. Replace it with good habits, ya know? Like taking a walk… taking a walk on the beach, ya know?* [Participant 1]

Other participants suggested discussing how to be ready “mentally” (2/10) and address emotions that may pose a challenge as they quit smoking (3/10).

*I think first you gotta prepare your mind mentally to wanna do it. It’s like with any addiction, you have to be willing to know that it’s bad for you and that it’ll end up killing you, and that you have to really be willing to let it go and turn it over. The mood swings and stuff, ya know? Because you do get moody when ya quit. I used to get really angry at everybody.* [Participant 1]

… *When I don’t have a cigarette I start getting antsy… and then I start that urge to like just, ya know. What’s the word… I get aggro! Put it that way.* [Participant 2]

Of note, only 1 participant mentioned wanting to know more about medications and stated that they wanted to know about the difference between nicotine replacement therapy and cigarettes. Additionally, only 2/10 individuals stated that they would like to hear more about the impact of smoking on their lungs. No other participants endorsed wanting to hear about health impacts, including health benefits of quitting. One participant reported that she did not want to hear about health detriments at all.

*I mean I’m already eat up with cancer so I don’t need to know anything about that, ya know?* [Participant 4]

## Discussion

### Principal Findings and Comparison With Previous Works

 In semi-structured interviews with cancer survivors who smoked cigarettes, participants indicated that they had previously attempted to quit smoking but had difficulty with making their abstinence last long-term. Participants reported numerous barriers, including routines or habits, other substance use, negative affect, and exposure to smoking in their social environment. This is consistent with previous literature indicating that alcohol use [17], depression [18-20], anxiety [21], and having more people who smoke in their social network [22] reduce the likelihood of someone successfully quitting smoking in non-cancer samples. Some findings are also consistent with previous research conducted among people with cancer. Specifically, individuals diagnosed with cancer with more depressive symptoms are more likely to continue smoking after diagnosis [23,24]. Additionally, among individuals with head and neck cancer, individuals who are exposed to smoking in their social environment (ie, peer or spousal smoking, others smoking at home) are less likely to achieve cessation [25]. Thus, addressing these barriers in smoking cessation interventions tailored for cancer survivors may be important.

When asked about preferences for future video-based interventions, participants largely endorsed not having preferences related to the gender presentation or age of the individual in the video. Participants may not be aware of a preference regarding age or gender of the presenter or do not wish to express that preference due to a social desirability bias. However, most participants did want to see someone with lived experience giving information. A recent systematic review and meta-analysis indicated that interventions that included peer support, particularly those led by individuals who had successfully quit smoking, seem to be helpful in improving smoking abstinence [26]. Additionally, videos created and aired by the Centers for Disease Control as part of the “Tips from Former Smokers” campaign that featured individuals who were affected by smoking-related diseases were effective in increasing awareness of health risks attributable to smoking [27]. It may be that having someone relatable in smoking cessation videos is important to make the information feel salient.

When asked about specific content they would like to see or topics they would like to learn more about, they had a difficult time generating ideas. Ideas that were presented varied and were largely related to behavioral strategies they could achieve on their own rather than information about medication or external resources. Survivors may not know where the gaps in their knowledge lie or may not be able to articulate the knowledge they do have regarding successful smoking cessation strategies that may be helpful for others. There may also be incorrect beliefs about smoking cessation strategies such as medication use that prevented participants from expressing interest in learning more, particularly as it relates to the safety or efficacy of medications like nicotine replacement therapy [28-30]. Previous studies have found that these concerns about nicotine replacement therapy act as a barrier to use [31,32]. While participants in the current study did not voice active opposition to learning more about medication options, misperceptions may have decreased their interest and providing accurate information about medications may increase interest in using them [33].

Overall, participants reported wanting to hear about actionable steps they could take to meet their goal of quitting smoking rather than hearing about the health implications. Many videos publicly available online do discuss the health impact or call for quitting smoking, but do not explain how to actually achieve this [11]. Thus, individuals may be aware that smoking is detrimental for health and can cause cancer, but given that they already have cancer, they may not see the relevance of hearing more about health implications of smoking. Indeed, cancer fatalism, or the belief that death is inevitable when someone has cancer, has been shown to be higher among individuals who smoke compared to those who do not [34], and higher cancer fatalism is associated with an increased likelihood of engaging in risky health behaviors such as smoking [35]. Higher cancer fatalism may also be associated with not seeking cancer-related health information [36]. Thus, it may be that participants do not wish to hear more about health implications of smoking due to fatalistic beliefs. It may be important to present possible health benefits to be gained with smoking cessation, even after a cancer diagnosis, to increase knowledge about the health benefits that come with smoking cessation.

### Limitations

** **While the current study has strengths such as expanding current knowledge about the unique needs and preferences of cancer for a novel tailored smoking cessation intervention, the current study has limitations worth noting. First, while the sample was diverse in terms of racial and gender identity, all participants were recruited from a cancer center at an academic medical center. Individuals receiving care from community clinics or health care providers in rural areas may have different preferences about content for videos for smoking cessation or the feasibility of watching these videos. It is also a small sample size, and may not be generalizable to all individuals with cancer who smoke. Additionally, participants were asked to recall quit attempts and barriers to cessation, which relied on patient memory. Participants may not have been able to accurately recall some of the resources they used to assist in smoking cessation attempts, or reasons for relapse. We also did not gather data related to education, income, or insurance status, which are important considerations in the context of smoking cessation. Lastly, the PI of the study conducted interviews and also served as one of the coders in the thematic analysis, which may introduce bias. However, all interviews were coded by two trained individuals to reduce the risk of bias.

### Conclusions

 Cancer survivors who smoke are open to novel intervention options such as videos that relay evidence-based information and would like to see peers with lived experience represented in these videos. While participants were able to clearly articulate their own experiences with and barriers to cessation, they had difficulty generating ideas about topics they would like to hear more about in future video interventions. Results from the current study provide considerations for researchers in the future who may be exploring the use of videos for smoking cessation in this vulnerable population. Information presented here, in combination with previously studied interventions and cessation guidelines, may be helpful when planning a video-based intervention. Videos presenting evidence-based information that guides actionable steps may be helpful to inform cancer survivors about quitting smoking while also being low touch and not resource-intensive. A clear next step is to use the current study results to create videos and test their acceptability, feasibility, and efficacy in changing smoking behaviors among cancer survivors, and explore optimal pathways for intervention delivery within current clinical or survivorship pathways.

## Supplementary material

10.2196/76792 Multimedia Appendix 1 Semi-structured interview guide used to facilitate telephone interviews.

## Abbreviations

MPH: Master of Public Health
PI: private investigator
TTS: tobacco treatment specialist
