# Supplementary material for: Thematic Analysis of Smoking Cessation and Future Cessation Interventions for Cancer Survivors: Convenience Sampling Study
Source: JMIR Form Res. 2025 Oct 23;9:e76792. doi: 10.2196/76792 (PMC12548965; doi:10.2196/76792)
Supplement: Multimedia Appendix 1 [file formative-v9-e76792-s001.docx]

**Interview Guide**

1. First, I just want to know a little about you. How old are you?
2. What type of cancer have you been diagnosed with?

PROBE: Have you had any other cancer diagnoses?

1. Let’s get started with some information about your smoking history. Please tell me a little about your smoking history, like if you are currently smoking or quit, how long you have been smoking, and how much.
2. How soon after you wake up do you/did you smoke your first cigarette?
3. Have you ever talked to a healthcare provider, like one of your doctors or nurses, about your smoking?

PROBE: Did you talk to providers here at MUSC as part of your cancer care team?

1. When were you first diagnoses with cancer? What was the diagnosis?

PROBE: What type of cancer, and what stage?

1. Have you ever tried to quit smoking? Tell me about any past quit attempts you’ve made.
2. What challenges, if any, did you face during your quit attempt? Were there any barriers to quitting?

PROBE: Did you feel you had the support and resources you needed to make a successful quit attempt?

1. Did you relapse after trying to quit?
2. (*If yes*) what happened when you relapsed to smoking? What were the triggers that made you return to smoking?
3. Have you ever used any medicine to help you quit smoking, such as Chantix (aka varenicline), Zyban (aka Wellbutrin), or nicotine replacement therapy options like patches of lozenges?

PROBE: (If endorsed use) What did you think about them?

1. Have you ever had counseling to help you quit smoking? How did that go? Who provided the counseling?
2. Have you ever watched videos about how to quit smoking?

PROBE: (If endorsed watching videos) How was this experience? Where did you watch these videos?

1. Do you think being able to watch videos on your personal device or computer about how to quit smoking could be helpful?

PROBE: (*If they do not have a computer or personal device*): Do you have somewhere else where you typically watch video-based content like tv shows or the news?

1. What information would you like to learn more about if you were to watch videos about quitting smoking?
2. How long do you think these videos should be?
3. How many videos would you be willing to watch while you were trying to quit?
4. Who would you want to be giving you the information in the video?

PROBE: A doctor or medical professional? A non-medical person like a peer or friend?

1. How would you want to receive these videos?

PROBE: Would you want a link texted to your phone or emailed to you, watching them during your healthcare appointments, or other ways?

1. Is there anything that could get in the way of you watching videos to help you quit smoking?
2. I also have just a couple of questions about your background and identity. Would you mind sharing your racial identity?
3. And what is your gender?
4. Thank you for taking time to talk with us today; this information is very helpful. Before we end, is there anything else you would like to share or any suggestions you would like to make?
